# 2093: Utilization of an ICD-coded electronic health records (EHR) database to characterize the epidemiology of prosopagnosia

**DOI:** 10.1017/cts.2017.93

**Published:** 2018-05-10

**Authors:** Christina Pressl, Caroline Jiang, Joel Correa da Rosa, Maximilian Friedrich, Winrich Freiwald, Jonathan Tobin

## Abstract

OBJECTIVES/SPECIFIC AIMS: We aim to examine the epidemiological characteristics of prosopagnosia by querying and analyzing a large deidentified clinical data set from 12 New York City-based hospitals and Federally Qualified Health Centers (FQHCs). The PCORI-funded New York City Clinical Data Research Network (NYC-CDRN) contains ~4.5 million deidentified ICD-coded electronic health records (EHRs) with comprehensive longitudinal information on demographics, patient visits, clinical conditions/diagnoses, laboratory and radiology results, medications, and clinical procedures. The NYC-CDRN will be expanded to include other data sources, including insurance claims, social determinant of health, patient reported outcomes, and patient generated data. The central hypothesis was that systematic mining of this database would reveal new epidemiological information about prosopagnosia. We developed a computable phenotype for prosopagnosia, using the International Classification of Diseases version 9 (ICD-9). The computable phenotype consisted of the diagnostic code for the condition under study, prosopagnosia (ICD-9 code 368.16), as well as the codes for known surrogate diagnoses. We expected to identify cases of acquired prosopagnosia, where the condition occurs only after brain damage, due to stroke, trauma, or meningitis for example, and cases of developmental prosopagnosia, where the condition is present from an early age, with no history of brain damage. The goals of this project were to provide new information about the condition’s prevalence rate in the New York City area, which could be furthermore translated into wider geographical areas and to yield novel details about its antecedents and comorbid conditions. METHODS/STUDY POPULATION: To determine the presence of the diagnosis of interest, prosopagnosia, and common co-occurring conditions among a New York City-based study population, we investigated a large database in collaboration with the NYC-CDRN. At the time the large database was mined it contained ~4 million ICD-9 coded EHRs. We first created a search paradigm; applicable for screening the database that consists of ICD-9 coded EHRs. We generated a list of ICD-9 codes indicative for the patients’ difficulties with the perception of faces (368.16), which indicates the presence of the condition as part of the psychophysical visual disturbances complex, and this code identified 871 patients. Furthermore, we collected codes that indicate the presence of conditions that are known to be surrogate diagnoses of prosopagnosia. ICD-9 codes for surrogate diagnoses included for example, 854.* (coding for personal history of traumatic brain injury, n=1,409), 434.01, 434.11, and 434.91 (coding for cerebral thrombosis, embolus and artery occlusion unspecified with cerebral infarction, n=19,409), and 191.2 (coding for malignant neoplasm of the temporal lobe, n=566). In October 2015, coding was changed to the new ICD-10 coding system. No additional patients were revealed from the data set when the cohort was searched for the presence of corresponding ICD-10 codes, as institutions are currently in transition from ICD-9 to ICD-10. Using this search query with the large database, we extracted novel information about the epidemiological and demographical distribution of prosopagnosia and furthermore, gained new knowledge about commonly associated diseases. The fact that it must be presumed that the majority of diagnoses of prosopagnosia have been made on the basis of patients’ self-reports and clinicians’ judgments represents a limiting factor in this study. We are currently exploring machine-learning strategies to identify potential false-negative cases among the patients with surrogate diagnoses. RESULTS/ANTICIPATED RESULTS: Investigations and application of our search query revealed a total number of n=129,549 patients carrying either the diagnosis code for prosopagnosia or the codes for the known surrogate diagnoses. There were 871 patients who carried the ICD-9 code 368.16, indicating the potential presence of prosopagnosia among other visual disturbances. Remaining patients (n=128,678) carried codes for known surrogate diagnoses, contained in the search query. Statistical analyses revealed elevated odds ratios for men (OR=1.55, 95% CI: 1.36, 1.77, *p*<0.0001), and for Black/African Americans Versus White individuals (OR=2.09, 95% CI: 1.74, 2.51, *p*<0.0001). DISCUSSION/SIGNIFICANCE OF IMPACT: Currently, the prevalence of prosopagnosia remains unknown. Face blind individuals are struggling to recognize their social contacts by their face only in every day life and are therefore prone to experience reduced quality of life. We searched the large NYC-based clinical database, containing more than 4.5 million deidentified ICD-coded health records, for cases of prosopagnosia to shed light into its prevalence and epidemiological characteristics. We furthermore, mined the database for cases carrying known surrogate diagnoses to explore the magnitude and characteristics of individuals potentially under increased risk. Our efforts address a great healthcare need, as they revealed new epidemiological knowledge of a vulnerable and understudied population. The results of this project reveal new insights into the epidemiological characteristics of prosopagnosia and its surrogate diagnoses, and demonstrate the feasibility of mining large clinical databases to identify rare clinical populations. Our results suggest the need for a more targeted diagnostic assessment of face perception abilities in populations under increased risk.

